# Vitreous Hemorrhage in Pediatric Age Group

**DOI:** 10.1155/2014/497083

**Published:** 2014-11-19

**Authors:** Dora H. AlHarkan, Eman S. Kahtani, Priscilla W. Gikandi, Ahmed M. Abu El-Asrar

**Affiliations:** ^1^Division of Pediatric Ophthalmology, King Khaled Eye Specialist Hospital, Riyadh, Saudi Arabia; ^2^Department and Division of Ophthalmology, College of Medicine, Qassim University, P.O. Box 4490, Buraidah, Qassim 51491, Saudi Arabia; ^3^Vitreoretinal Division, King Khaled Eye Specialist Hospital, P.O. Box 7191, Riyadh 11462, Saudi Arabia; ^4^Department of Ophthalmology, King Abdulaziz University Hospital, Old Airport Road, P.O. Box 245, Riyadh 11411, Saudi Arabia; ^5^Dr. Nasser Al-Rashid Research Chair in Ophthalmology, Riyadh, Saudi Arabia

## Abstract

*Purpose.* To identify and study causes of vitreous hemorrhage (VH) in pediatric age group and to investigate factors predicting visual and anatomical outcomes. *Procedure.* A retrospective review of patients aged 16 years or less with the diagnosis of vitreous hemorrhage from January 2005 until December 2010. *Results.* A total number of 230 patients (240 eyes) were identified. Traumatic vitreous hemorrhage accounted for 82.5%. In cases of accidental trauma, final visual acuity of 20/200 was significantly associated with visual acuity of ≥20/200 at presentation and the absence of retinal detachment at last follow-up. Patients with nontraumatic vitreous hemorrhage were significantly younger with higher rates of enucleation/evisceration/exenteration and retinal detachment at last follow-up compared to traumatic cases. *Conclusion.* Trauma is the most common cause of VH in pediatric age group. In this group, initial visual acuity was the most important predictor for visual outcome, and the presence of retinal detachment is a negative predictor for final good visual outcome. The outcome is significantly worse in nontraumatic cases compared to traumatic cases.

## 1. Introduction

Vitreous hemorrhage has been well investigated in the adult age group [1–5]. In children, however, vitreous hemorrhage is uncommon, and as a result, it is not studied as well as in the adult age group and little is known about it. On literature review, different causes have been published, but most of these publications are either case reports or a small series describing particular group of patients or different modalities of treatment [6–26]. To the best of our knowledge, there are only two studies that investigated vitreous hemorrhage as a separate entity in children in English literature and these studies showed different causes than that published in adults [27, 28]. In these two studies trauma was the most common cause of vitreous hemorrhage in children accounting for 73.1% [28] and 68.5% [27]. Ocular trauma is the leading cause of monocular visual disability and monocular blindness in children. Vitreous hemorrhage has been reported in different studies (either in children alone or in both adult and children) as one of poor visual prognostic signs in both open globe injury [29–33] and closed globe injury [34]. In a large series of ocular trauma in children, vitreous hemorrhage was documented in 6.7% of the eyes [35]. Another study reported VH in 20% of eyes after open globe injury in children [36]. Traumatic vitreous hemorrhage in children can lead to tractional retinal detachment and occlusion amblyopia [34, 37]. Spirn et al. reported that the most common cause of nontraumatic vitreous hemorrhage in children was regressed retinopathy of prematurity (ROP) accounting for 5.9% [28]. In a recent study from India, Rishi et al. demonstrated that idiopathic retinal periphlebitis was the most common cause in eyes with nontraumatic vitreous hemorrhage in children accounting for 12.6% [27]. Because little is known about vitreous hemorrhage in children, we conducted this retrospective study to investigate etiology and visual and anatomic outcomes of vitreous hemorrhage in pediatric age group.

## 2. Patient and Methods

Institutional board approval was obtained for this project. We retrospectively reviewed all charts of patients aged 16 years or less and diagnosed with vitreous hemorrhage, seen at King Khaled Eye Specialist Hospital, Riyadh, Saudi Arabia, from of January 2005 until December 2010. Charts were identified through the coding system. Our inclusion criteria included any patient diagnosed with vitreous hemorrhage either in the outpatient clinic note or in the operative note, with the age of sixteen years or less. Exclusion criteria included follow-up duration of less than one month and the development of endophthalmitis at presentation or during the follow-up. A specialized data collection sheet was designed to include the following: demographic data (age, gender, date of birth, and laterality), presentation (referral, emergency case, or postoperatively), history, and causes of vitreous hemorrhage. History of recent surgery as well as birth trauma was obtained. In traumatic cases, trauma was classified as closed globe injury (contusion, lamellar laceration) or open globe injury (perforation, penetration, rupture, with or without intraocular foreign body) [38]. The mechanism of trauma was identified (sharp, blunt, fireworks, or missile). In addition the following information was collected: vision at presentation, the presence of strabismus or nystagmus, the presence of an afferent pupillary defect, cornea status, extent of the wound (≤10 mm or >10 mm), zone of Injury (zone I: cornea only, zone II: corneoscleral within 5 mm of the limbus, and zone III: corneoscleral extending >5 mm from the limbus) [33], presence of hyphema, lens status, density of vitreous hemorrhage based on indirect ophthalmoscopy (mild to moderate if there was a view to the fovea and blood vessels or severe if there was no view to the fovea and blood vessels), condition of the retina either flat or detached, B-scan findings, management that was done, follow-up duration, and clinical findings at last follow-up. The diagnosis of the cause of nontraumatic vitreous hemorrhage was based on the clinical diagnosis written in the chart of the patient.

Data were analyzed first to identify the causes of vitreous hemorrhage. Univariate analysis was used to identify factors that predicted the visual and anatomical outcomes in traumatic cases by studying the following variables: age of the patient, initial vitreous hemorrhage, mechanism of injury, wound length, wound location, lens injury at presentation, iris injury, hyphema, density of vitreous hemorrhage, retinal detachment at presentation, optic nerve avulsion, vitrectomy performed, lens status on last follow-up (clear, cataract, pseudophakic, or aphakic), and the presence of retinal detachment. The same factors were used to determine factors affecting the anatomical outcome. Then we compared traumatic and nontraumatic cases of vitreous hemorrhage.

## 3. Statistical Methods

Comparisons between two categorical variables were made using the Chi-square test or Fisher's exact test, as appropriate. Student's *t*-test was used to compare the difference between two proportions from the same sample. A *P* value less than 0.05 indicated statistical significance. Stepwise logistic regression analysis was conducted to identify the variables that tended to influence the achievement of final visual acuity of 20/200 or better after adjustment for all other factors. A 95% confidence interval (CI) around the odds ratio that did not include a value of 1.0 indicated statistical significance. The statistical software used for the analyses included SPSS Version 15.0, Stats Direct Version 1.9, and BMDP 2007.

## 4. Results

During the study period 230 patients (240 eyes) met our inclusion criteria and were included in the study. Causes of vitreous hemorrhage were classified into traumatic and nontraumatic (Table 1). Traumatic vitreous hemorrhage accounted for 198 out of 240 (82.5%) eyes. Of these 198 eyes, 11 developed vitreous hemorrhage after surgical intervention (nonaccidental trauma) (7 eyes after glaucoma surgeries, 2 eyes after secondary intraocular lens implantation, and 2 eyes after retinal detachment repair). Nontraumatic cases accounted for 42 out of 240 (17.5%) eyes.  Table 2 shows the demography of these patients.

### 4.1. Nontraumatic Vitreous Hemorrhage

**Figure 1:**
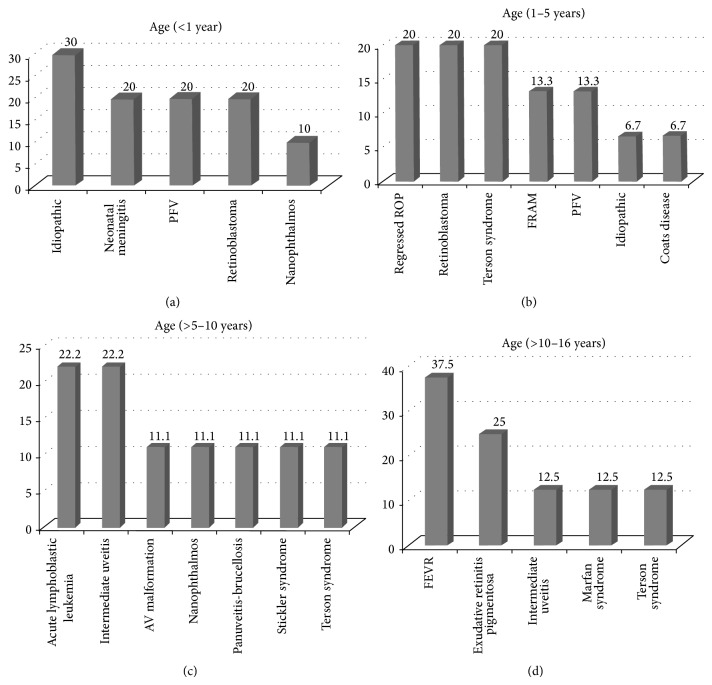
Causes of nontraumatic vitreous hemorrhage according to age group. (a) PFV: persistent fetal vasculature. (b) ROP: retinopathy of prematurity. FRAM: familial retinal artery macroaneurysm. PFV: persistent fetal vasculature. (c) AV: arteriovenous. (d) FEVR: familial exudative vitreoretinopathy.

Causes of nontraumatic vitreous hemorrhage are illustrated in Table 1. This group was divided into four subgroups according to the age at presentation, (Table 3). In the group of patients aged less than one year, spontaneous idiopathic cause was the most common cause (Figure 1(a)). In the group of patients aged more than one year up to 5 years, spontaneously regressed retinopathy of prematurity (ROP), retinoblastoma (RB), and Terson's syndrome were the most common causes (Figure 1(b)). In the group of patients aged more than 5 years up to 10 years, acute lymphoblastic leukemia and intermediate uveitis were the most common causes (Figure 1(c)). In the group of patients aged more than 10 years up to 16 years, familial exudative vitreoretinopathy (FEVR) and exudative retinitis pigmentosa were the most common causes (Figure 1(d)). All patients with Terson's syndrome were related to head trauma and had a history of intensive care unit admission. Three patients had history of subdural hematoma and one patient had history of cerebral hematoma. The mean follow-up for nontraumatic VH cases was 25.8 ± 22.1 months, the median was 16 months, and the range was 2–75 months.  Table 4 is a summary of the clinical findings at presentation, management, and the outcome of this group of patients.

Observation was the modality of management for the nontraumatic cases in 19 out of 42 (45.2%) eyes. Incisional management was the modality of treatment done for 17 out of 42 (40.5%) eyes that included vitrectomy with or without tamponade or retinal detachment surgery and enucleation/exenteration. Vitrectomy was done for 12 out of 42 (28.6%) eyes and enucleation/exenteration was done for 5 out of 42 (11.9%) eyes. In addition, laser therapy was the only modality of treatment done for 6 out of 42 (14.3%) eyes.

#### 4.1.1. Visual Outcome of Nontraumatic Vitreous Hemorrhage

The vision was available at presentation and at last follow-up in only 28 out of 42 (66.7%) eyes. At presentation the vision was 20/200 or more in 8 out of 28 (28.6%) eyes, counting fingers in 4 out of 28 (14.3%) eyes, and light perception to hand motions in 16 out of 28 (57.1%) eyes. At last follow-up the vision was 20/200 or more in 15 out of 28 (53.6%) eyes, counting fingers in 3 out of 28 (10.7%) eyes, and light perception to hand motion in 10 out of 28 (35.7%) eyes. The distribution of initial and final visual acuity is illustrated in Table 5. We found no change in visual acuity in 16 out of 28 (57.1%) eyes. The vision improved in 10 out of 28 (35.7%) eyes. The vision deteriorated in 2 out of 28 (7.2%) eyes. The prevalence of poor vision (counting fingers or less) decreased from 20 out of 28 (71.4%) eyes at presentation to 13 out of 28 (46.4%) eyes at last follow-up, but the difference between the two percentages was not statistically significant (*P* = 0.2215; Student's *t*-test for comparing two percentages from the same sample). The prevalence of visual acuity of 20/200 or more increased from 8 out of 28 (28.6%) eyes at presentation to 15 out of 28 (53.6%) eyes at last follow-up, but the difference between the two percentages was not statistically significant (*P* = 0.0573; Student's *t*-test for comparing two percentages from the same sample).

Univariate analysis was performed to investigate if the density of vitreous hemorrhage or the performance of incisional surgery (in the form of pars plana vitrectomy) would predict the visual outcome (Table 6). No significant correlation was found with either factor.

#### 4.1.2. Anatomical Outcome of Nontraumatic Vitreous Hemorrhage

Retinal detachment at presentation was diagnosed in 14 out of 42 (33.3%) eyes. Five eyes had rhegmatogenous retinal detachment (RRD), 4 eyes had tractional retinal detachment (TRD), 4 eyes had combined RRD and TRD, and one eye had exudative retinal detachment. Causes of retinal detachment included retinoblastoma in 4 eyes, FEVR in two eyes, neonatal meningitis in two eyes, Coats' disease in one eye, familial retinal arterial macroaneurysm (FRAM) in one eye [39, 40], Marfan syndrome in one eye, Stickler syndrome in one eye, Retinitis pigmentosa (RP) with Coats-like disease in one eye, and nanophthalmia in one eye. At last follow-up 11 out of 42 (26.2%) eyes had persistent retinal detachment. Two eyes with Terson's syndrome had inoperable RD, one eye with persistent fetal vasculature had inoperable TRD, one eye with FEVR had recurrent RD, one eye with FRAM had inoperable TRD, two eyes with neonatal meningitis had inoperable RD, one eye with Coats disease had inoperable RD, one eye with Marfan syndrome had recurrent RD, one eye with nanophthalmos had inoperable RD, and one eye with Stickler syndrome had inoperable RD.  Table 4 summarizes the management that was done for each case.

### 4.2. Accidental Traumatic Vitreous Hemorrhage

The mechanism of accidental trauma was available in 174 out of the 187 charts. The most common mechanism of accidental trauma was trauma by sharp object accounting for 99 out of 174 (56.9%) eyes followed by blunt trauma accounting for 61 out of 174 (35.1%) eyes, and trauma by missile and fireworks, each accounting for 7 out of 174 (4%) eyes. Intraocular foreign body was found in 8 traumatized eyes. In cases of accidental trauma caused by blunt trauma, 47 (77%) patients were males and the mean age was 8.78 ± 4.26 (range: 0.33–16) years. The mean follow-up for accidental traumatic VH cases was 23.8 ± 18.9 months, the median was 20 months, and the range was 1–75 months.

**Figure 2:**
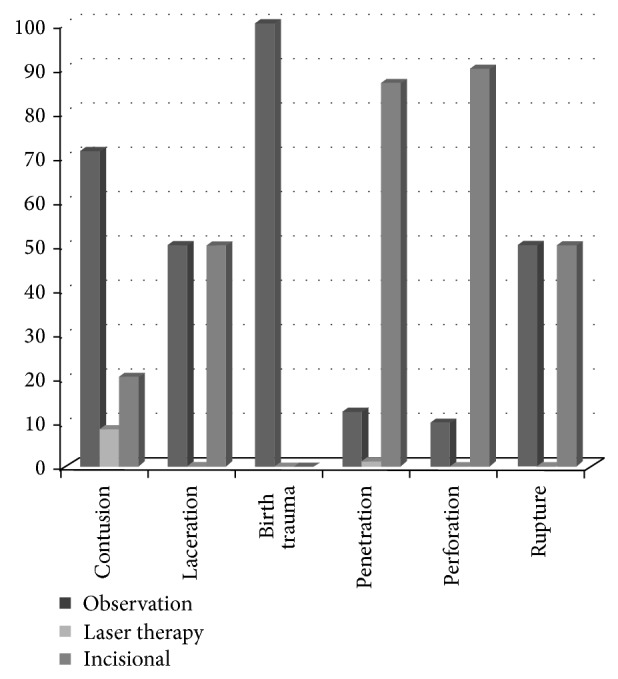
Management modalities used in different types of vitreous hemorrhage caused by accidental trauma.

Vitrectomy was done for 104 out of 187 (55.6%) eyes, and evisceration/enucleation was done for 3 out of 187 (1.6%) eyes. Observation was the modality of treatment for 64 out of 187 (34.2%) eyes. Prophylactic laser photocoagulation was performed in 6 out of 187 (3.2%) eyes that presented with retinal break without RD (Figure 2).

#### 4.2.1. Visual Outcome of Traumatic Vitreous Hemorrhage after Accidental Trauma

Visual acuity at presentation and last follow-up was available for 133 (71.1%) of accidentally traumatized eyes out of 187. The vision at presentation was 20/200 or more in 33 out of 133 (24.9%) eyes, counting fingers in 26 out of 133 (19.5%) eyes, and light perception to hand motions in 74 out of 133 (55.6%) eyes. At last follow-up the vision was 20/200 or more in 81 out of 133 (60.9%) eyes, counting fingers in 22 out of 133 (16.5%) eyes, and light perception to hand motions in 30 out of 133 (22.6%) eyes. The distribution of initial and final visual acuity is illustrated in Table 7. The vision did not change in 68 out of 133 (51.1%) eyes. The vision improved in 61 out of 133 (45.9%) eyes. The vision deteriorated in 4 out of 133 (3%) eyes. The prevalence of poor vision (counting fingers or less) significantly reduced from 100 (75.2%) eyes at presentation to 52 (39.1%) eyes at last follow-up (*P* ≤ 0.001; Student's *t*-test for comparing two percentages from the same sample). Prevalence of visual acuity of 20/200 increased more significantly from 33 (24.9%) eyes at presentation to 81 (60.9%) at last follow-up (*P* ≤ 0.001; Student's *t* test for comparing two percentages from the same sample).

Univariate analysis was performed to investigate factors predicting the visual outcome. It demonstrated a significant association between final visual acuity of 20/200 or better and the following factors: initial visual acuity of 20/200 or better (*P* < 0.001), mild to moderate degree of VH at presentation (partial view to the retina) (*P* = 0.0057), and presence of clear lens or pseudophakia at last follow-up (*P* < 0.001) (Table 8).

Univariate analysis was performed to identify factors associated with poor final visual outcome (hand motions or less). Poor final visual outcome was significantly associated with poor initial visual acuity (hand motions or less) (*P* < 0.001), age at presentation of 3 years or younger (*P* = 0.0033), severe vitreous hemorrhage at presentation (no view to the fundus) (*P* = 0.0039), and presence of cataract or aphakia at last follow-up (*P* = 0.0012) (Table 8).

Multivariate analysis based on stepwise logistic regression analysis was conducted to identify the variables that tended to influence the attainment of final visual acuity of 20/200 or better after adjustment for all the other factors. In this analysis, all the variables that were investigated for association with final visual acuity in univariate analysis were included as the predictor or independent variables. Visual acuity of 20/200 or better at presentation was significantly positively associated with the attainment of final visual acuity of 20/200 or better at last follow-up (odds ratio = 9.08; 95% confidence interval [CI] = 2.46–33.5). On the other hand, presence of retinal detachment at last follow-up was hindrance to achieving final visual acuity of 20/200 or better (odds ratio = 0.24; 95% CI = 0.09–0.68).

#### 4.2.2. Anatomical Outcome of Traumatic Vitreous Hemorrhage after Accidental Trauma

Retinal detachment was seen in 41 out of 187 (21.9%) eyes of traumatic vitreous hemorrhage at presentation. In addition, 8 (4.28%) eyes developed retinal detachment during follow-up. At last follow-up 18 (9.6%) eyes had persistent retinal detachment. These included nine patients with persistent RD and nine patients developed new RD during follow-up.  Table 9 summarizes the clinical data of these 18 patients.

On univariate analysis, retinal detachment at final follow-up was significantly associated with presenting age of 3 years or younger (*P* = 0.0209), eyes that had trauma caused by open globe injury (*P* = 0.0341), eyes that underwent vitrectomy (*P* = 0.043), and presence of aphakia at last follow-up (0.0013) (Table 10). Multivariate analysis to identify the variables that tended to influence the anatomical outcome could not be done due to small number of the affected eyes.

### 4.3. Comparison between Nontraumatic Vitreous Hemorrhage and Vitreous Hemorrhage Caused by Accidental Trauma

The mean age of accidental traumatic vitreous hemorrhage cases was significantly higher than in nontraumatic cases (7.7 years versus 4.9 years, resp.; *P* = 0.0004). Males were the most affected in both traumatic 134 out of 187 (71.7%) patients and 24 nontraumatic cases out of 42 (57.1%) patients. The rate of performance of vitrectomy was significantly higher in eyes with accidental trauma (55.6%) than in nontraumatic (28.6%) (*P* = 0.0027). The rate of enucleation/evisceration/exenteration was significantly higher in nontraumatic eyes than in eyes with accidental trauma (11.9% versus 1.6%; *P* = 0.0067). The incidence of retinal detachment at last follow-up was significantly higher in nontraumatic (42.9%) than in eyes with accidental trauma (2.1%) (*P* = 0.0159) (Table 11).

## 5. Discussion

In this retrospective study of pediatric vitreous hemorrhage that presented to our institute, we analyzed 240 eyes of 230 children diagnosed with vitreous hemorrhage. The causes of vitreous hemorrhage were classified as traumatic and nontraumatic causes. Trauma was the most common cause of vitreous hemorrhage in children accounting for 82.5%. Nontraumatic cases accounted for 17.5%. These findings are in agreement with previous studies by Spirn et al. [28] and Rishi et al. [27] who demonstrated that trauma was the most common cause of vitreous hemorrhage in pediatric age group (73.1% and 68.5%, resp.).

In our study, the etiology on nontraumatic vitreous hemorrhage was in agreement with other reported series [27, 28]. However, in the present study, retinoblastoma was more frequent compared to other studies. Eyes with retinoblastoma had the worst prognosis compared with other nontraumatic causes. Previous studies showed that vitreous hemorrhage in eyes with retinoblastoma is rare and its presence is a predictor of poor prognosis [41, 42]. Shields et al. [43] recommend that the clinician should seriously consider the possibility of retinoblastoma in children who present with signs of unexplained vitreous hemorrhage or endophthalmitis, even if they are older than 5 years of age.

The novel finding in our study is that nontraumatic vitreous hemorrhage occurred in younger age group and had poor prognosis in the form of high incidence of retinal detachment and enucleation/exenteration as compared with traumatic cases. This can be explained by the fact that many of nontraumatic causes like hereditary diseases or retinoblastoma tend to occur in younger age group, which are usually associated with retinal detachment, while trauma tends to occur in a physically active child.

In the present study, in traumatic cases, the most common mechanism of trauma was trauma by a sharp object followed by blunt trauma. Our findings are in contrast to other studies that reported that blunt trauma was the most common mechanism of traumatic vitreous hemorrhage in pediatric age group [27, 28]. Vitreous hemorrhage due to shaken-baby syndrome was reported in previous studies with an incidence of 8.6% [28]. However, in the present retrospective study we did not encounter any case of shaken-baby syndrome. This can be attributed to the fact that our hospital is a specialized eye hospital and patients who are referred to us should have stable medical condition. It is known that babies with shaken-baby syndrome usually presented with unstable medical condition [19].

In this study, univariate analysis of cases that presented after accidental trauma demonstrated that the initial visual acuity of 20/200 or better was a significant important prognostic indicator of final visual outcome of 20/200 or more that remained a significant predictor by using logistic regression analysis. On the other hand, we found that worse initial visual acuity of hand motions or less was associated with a poor visual outcome (hand motions or less). Our observation is consistent with previous studies that included children alone [36] or both children and adults [29, 33, 44–47]. In addition, our study showed a significant association between younger age at presentation (3 years or less) and poor visual outcome and this finding was similar to other published studies [36, 48]. Amblyopia is expected to be the cause of poor vision in this younger age group.

Our study showed that density of vitreous hemorrhage at presentation significantly affects the visual outcome. Eyes with dense vitreous hemorrhage with no view to the fundus had a poor visual outcome. On the other hand, eyes with mild to moderate vitreous hemorrhage (partial view to the retina) had a significant association with visual outcome of 20/200 or better. This is similar to Matthews and Das finding in infants with shaken-baby syndrome who developed vitreous hemorrhage [19]. Our analysis demonstrated that retinal status significantly affects the visual outcome. In addition, we found that lens status at last follow-up significantly affected the visual outcome. Presence of clear lens or pseudophakia was associated with visual outcome of 20/200 or more and the presence of aphakia or cataract was associated with poor visual outcome. This finding is consistent with previous reports [29, 33, 36].

In this study, we also investigated factors that predict final anatomical outcome. Our analysis showed that the development of retinal detachment in eyes with vitreous hemorrhage caused by accidental trauma was significantly associated with younger age at presentation, open globe injury, performance of vitrectomy, and aphakia at last follow-up. Our findings are consistent with other reports that demonstrated that the risk of retinal detachment is high in aphakic eyes [49] and eyes that had vitrectomy [29].

A major limitation of our study is its retrospective nature. Therefore, in most of the patients included in this study, factors related to amblyopia were not investigated. The visual outcome in children with vitreous hemorrhage especially those aged 8 years or less is highly dependent on the duration of vitreous hemorrhage, early initiation of amblyopia treatment, clarity of visual axis, and also the family compliance with amblyopia treatment and wearing of glasses.

In conclusion, etiology of vitreous hemorrhage in children is unique. Trauma is the most common cause of vitreous hemorrhage in pediatric age group. In this group, initial visual acuity was the most important predictor for visual outcome, and the presence of retinal detachment at the final follow-up was a negative predictor for final good visual outcome. Patients with nontraumatic vitreous hemorrhage were significantly younger and the visual outcome was significantly worse in comparison to patients with traumatic vitreous hemorrhage.

## Figures and Tables

**Table 1:** Causes of vitreous hemorrhage.

Cause	Number of eyes (%)
Traumatic	198 (82.5%)
(i) Closed globe injury	63 (26.3%)
(a) Contusion	59 (24.6%)
(b) Laceration	2 (0.8%)
(c) Birth trauma	2 (0.8%)
(ii) Open globe injury	124 (51.7%)
(a) Penetration	104 (43.3%)
(b) Perforation	10 (4.2%)
(c) Rupture	10 (4.2%)
(iii) Postoperative	11 (4.6%)

Nontraumatic	42 (17.5%)
Retinoblastoma (RB)	5 (2.1%)
Terson's syndrome	5 (2.1%)
Persistent fetal vasculature (PFV)	4 (1.7%)
Idiopathic	4 (1.7%)
Regressed retinopathy of prematurity (ROP)	3 (1.3%)
Familial exudative vitreoretinopathy (FEVR)	3 (1.3%)
Intermediate uveitis	3 (1.3%)
Associated with acute lymphoblastic leukemia	2 (0.8%)
Retinitis pigmentosa (RP) with Coats-like disease	2 (0.8%)
Familial retinal artery macroaneurysm (FRAM)	2 (0.8%)
Nanophthalmos	2 (0.8%)
Neonatal meningitis	2 (0.8%)
Panuveitis (brucellosis)	1 (0.4%)
Stickler diseases with RRD	1 (0.4%)
Arteriovenous malformation	1 (0.4%)
Coats disease	1 (0.4%)
Marfan syndrome with RRD	1 (0.4%)

RRD: rhegmatogenous retinal detachment.

**Table 2:** Demographic features of 230 patients (240 eyes) with vitreous hemorrhage (%).

Age (in years)	
(i) Mean ± SD	7.5 ± 4.6
(ii) Median	7.4
(iii) Range	0–16
Duration of FU (in months)	
(i) Mean ± SD	24.65 ± 19.08
(ii) Median	20
(iii) Range	1–75
Gender (number of eyes (%))	
(i) Male	164 (71.30)
(ii) Female	66 (28.70)
Laterality (number of patient (%))	
(i) Unilateral	220 (95.65)
(ii) Bilateral	10 (4.35)
Involved eye (number of eyes (%))	
(i) Right	118 (49.17)
(ii) Left	122 (50.83)

SD: standard deviation. FU: follow-up.

**Table 3:** Causes of vitreous hemorrhage for nontraumatic eyes according to the age group.

Causes	Age groups				Total *N* (%)
	<1 year *N* (%)	1–5 years *N* (%)	>5–10 years *N* (%)	>10 years *N* (%)	
Retinoblastoma	2 (20.0)	3 (20.0)	0 (0.0)	0 (0.0)	5
Terson's syndrome	0 (0.0)	3 (20.0)	1 (11.1)	1 (12.5)	5
Spontaneous/idiopathic	3 (30.0)	1 (6.7)	0 (0.0)	0 (0.0)	4
PFV	2 (20.0)	2 (13.3)	0 (0.0)	0 (0.0)	4
Spontaneous/regressed ROP	0 (0.0)	3 (20.0)	0 (0.0)	0 (0.0)	3
FEVR	0 (0.0)	0 (0.0)	0 (0.0)	3 (37.5)	3
Intermediate uveitis	0 (0.0)	0 (0.0)	2 (22.2)	1 (12.5)	3
Acute lymphoblastic leukemia	0 (0.0)	0 (0.0)	2 (22.2)	0 (0.0)	2
Exudative retinitis pigmentosa	0 (0.0)	0 (0.0)	0 (0.0)	2 (25.0)	2
FRAM	0 (0.0)	2 (13.3)	0 (0.0)	0 (0.0)	2
Nanophthalmos	1 (10.0)	0 (0.0)	1 (11.1)	0 (0.0)	2
Neonatal meningitis	2 (20.0)	0 (0.0)	0 (0.0)	0 (0.0)	2
AV malformation	0 (0.0)	0 (0.0)	1 (11.1)	0 (0.0)	1
Coats disease	0 (0.0)	1 (6.7)	0 (0.0)	0 (0.0)	1
Marfan syndrome with RRD	0 (0.0)	0 (0.0)	0 (0.0)	1 (12.5)	1
Panuveitis-brucellosis	0 (0.0)	0 (0.0)	1 (11.1)	0 (0.0)	1
Stickler syndrome RRD	0 (0.0)	0 (0.0)	1 (11.1)	0 (0.0)	1
Total	**10 (23.8)**	**15 (35.7)**	**9 (21.4)**	**8 (19.0)**	**42 (100)**

ROP: regressed retinopathy of prematurity. AV: arteriovenous. FRAM: familial retinal artery macroaneurysm. FEVR: familial exudative vitreoretinopathy. PFV: persistent fetal vasculature. RRD: rhegmatogenous retinal detachment.

**Table 4:** Summary of the causes of nontraumatic vitreous hemorrhage.

Diagnosis	Eyes (Pt)	Age	Gender	Presentation	VA presenting	Management	Outcome
(1) Retinoblastoma	5 (5)	8 m 9 m 3 Y 3 Y 4 Y	2 M 3 F	Leukocoria (2) Strabismus (2) Seen in clinic postradiation therapy (1)	NA (2) No F&F (1) CFNF (1) LP (1)	Enucleation (4) Exenteration (1)	Prosthesis (5)
(2) Terson's syndrome	5 (4)	11 Y 6 Y 4 Y 1 Y	4 M	Decreased vision (3) Strabismus (1)	20/200 (1) F&F (3) HM (1)	PPV (4) Observation (I)	20/25 (1) 20/80 (1) F&F (1) No F&F, inoperable RD (2)
(3) PFV	4 (3)	1 Y 5 m 4 m	1 M 2 F	Leukocoria (3) Small eye (1)	Not F&F (4)	Observation (3) Prosthesis (1)	20/100 (2) Prosthesis (1) No F& F, inoperable TRD (1)
(4) Idiopathic	4 (3)	4 Y 3 m 3 m	2 M 1 F	Strabismus (1) Behavioral (2) changes	No F&F (3) NA (1)	PPV (4)	6/30 (1) 20/80 (2) NA (1)
(5) Regressed ROP	3 (3)	3 Y 3 Y 5 Y	2 M 1 F	Decreased vision (3)	NA (2) No F&F (1)	PPV (2) Observation (I)	F&F (1) 10/30 (1) 20/125 (1)
(6) FEVR	3 (2)	11 Y 13 Y	1 M 1 F	Decreased VA (3)	20/40 (1) 20/30 (1) 20/70 (1)	PPV (1) Laser therapy (2)	HM, RD (1) 20/30 (1) 20/25 (1)
(7) Intermediate uveitis	3 (2)	7 Y 9 Y 11 Y	2 M	Decreased vision (2) Seen in FU (1)	HM (1) CFNF (1) 1/200 (1)	PPV (1) Observation (2)	20/30 (1) 20/40 (1) 20/20 (1)
(8) FRAM	2 (1)	2 Y	1 M	Strabismus (1) Decreased vision (2)	F&F (1) No F&F (1)	Laser therapy (2)	20/30 (1) 4/200, inoperable TRD (1)
(9) Retinitis pigmentosa with Coats-like disease	2 (1)	13 Y	1 F	Decreased vision (2)	20/50 (1) 20/60 (1)	Laser therapy (2)	20/40 (1) 20/300 (1)
(10) Neonatal meningitis	2 (1)	6 m	1 M	Behavioral changes (2)	No F&F (2)	Observation (2)	No F&F, both inoperable TRD + RRD (2)
(11) Nanophthalmos	2 (2)	6 Y 3 m	1 M 1 F	Strabismus (1) Leukocoria (1)	No F&F (2)	Observation (2)	NLP, inoperable RRD + TRD (1) F&F (1)
(12) Associated with ALL	2 (1)	10 Y	1 F	Strabismus (2) Decreased (2) vision	CF1F (1) HM (1)	Observation (2)	3/200 (1) 4/200 (1)
(13) Panuveitis	1 (1)	6 Y	1 M	Decreased vision	20/200	Observation	20/30
(14) AV malformation	1 (1)	9 Y	1 F	Decreased vision	20/400	Laser	20/300
(15) Coats disease	1 (1)	3 Y	1 M	Strabismus and Hx laser therapy	No F&F	Observation	NA, inoperable TRD + RRD
(16) Marfan syndrome + RRD	1 (1)	11 Y	1 F	1 decreased vision	CF2F	PPV	LP, inoperable RD
(17) Stickler syndrome + RRD	1 (1)	6 Y	1 F	Redness	No F&F	Observation	NLP vision, inoperable RD

VA: visual acuity. Pt: patient. m: month. Y: year. M: male. F: female. NA: not available. F&F: fix and follow. CFNF: counting finger near to face. LP: light perception. HM: hand motion. PPV: pars plana vitrectomy. RD: retinal detachment. RRD: rhegmatogenous retinal detachment. TRD: tractional retinal detachment. PFV: persistent fetal vasculature. ROP: retinopathy of prematurity. FEVR: familial exudative vitreoretinopathy. FU: follow-up. FRAM: familial retinal artery macroaneurysm. NLP: no light perception. ALL: acute lymphoblastic leukemia. CF1F: counting finger one foot. AV: arteriovenous. Hx: history. CF2F: counting finger two feet.

**Table 5:** The distribution of initial and final visual acuity for eyes with nontraumatic vitreous hemorrhage.

VA at last follow-up	VA at presentation			Total
	LP-HM	CF	≥20/200	
≥20/200	6	2	7	15 (53.6%)
CF	2	1	0	3 (10.7%)
LP-HM	8	1	1	10 (35.7%)

Total	16 (57.1%)	4 (14.3%)	8 (28.6%)	28 (100%)

LP: light perception. HM: hand motion. CF: counting finger.

**Table 6:** Factors predicting visual acuity at last follow-up for eyes with nontraumatic vitreous hemorrhage. Vision was available in 33 out of 42 eyes.

Variable	Visual acuity at last follow-up		
	LP-HM (%)	CF (%)	≥20/200 (%)
(1) Density of VH^*^			
(i) Partial view (*N* = 12)	3 (25.0)	3 (25.0)	6 (50.0)
(ii) No view (*N* = 19)	6 (31.6)	3 (15.8)	10 (52.6)
*P* value	**0.991**	**0.869**	**0.820**

(2) PPV performed			
(i) Yes (*N* = 10)	3 (30.0)	1 (10.0)	6 (60.0)
(ii) No (*N* = 23)	8 (34.8)	5 (21.7)	10 (43.5)
*P* value	**0.908**	**0.725**	**0.599**

LP: light perception. HM: hand motion. CF: counting finger. *N*: number. VH: vitreous hemorrhage. PPV: pars plana vitrectomy. FU: follow-up. ^*^Missing data: 2 cases.

**Table 7:** The distribution of initial and final visual acuity in eyes with vitreous hemorrhage caused by accidental trauma.

VA at last follow-up	VA at presentation			Total
	LP-HM	CF	≥20/200	
≥20/200	35	16	30	81 (60.9%)
CF	10	9	3	22 (16.5%)
LP-HM	29	1	0	30 (22.6%)

Total	74 (55.6%)	26 (19.5%)	33 (24.9%)	133 (100%)

LP: light perception. HM: hand motion. CF: counting finger.

**Table 8:** Factors predicting visual acuity at last follow-up for eyes with vitreous hemorrhage caused by accidental trauma. Vision was available in 165 out of 187 eyes.

Variable	Visual acuity at last follow-up		
	LP-HM (%)	CF (%)	≥20/200 (%)
(1) VA at presentation			
(i) LP-HM (*N* = 74)	29 (39.2)	10 (13.5)	35 (47.3)
(ii) CF (*N* = 26)	1 (3.8)	9 (34.6)	16 (61.6)
(iii) ≥20/200 (*N* = 33)	0 (0.0)	3 (9.1)	30 (90.9)
*P* value	**<0.001** ^*^	**0.0312** ^*^	**<0.001** ^*^
(2) Age at presentation			
(i) 0–3 years (*N* = 24)	12 (50.0)	2 (8.3)	10 (41.7)
(ii) 4–8 years (*N* = 66)	16 (24.2)	11 (16.7)	39 (59.1)
(iii) 9–16 years (*N* = 75)	12 (16.0)	15 (20.0)	48 (64.0)
*P* value	**0.0033** ^*^	**0.4394**	**0.1536**
(3) Type of trauma			
(i) Open globe (*N* = 108)	29 (26.9)	19 (17.6)	60 (55.5)
(ii) Closed globe (*N* = 57)	11 (19.3)	9 (15.8)	37 (64.9)
*P* value	**0.3758**	**0.9399**	**0.3198**
(4) Mechanism of trauma			
(i) Blunt (*N* = 53)	10 (18.9)	11 (20.8)	32 (60.3)
(ii) Sharp/missile (*N* = 94)	26 (27.7)	15 (16.0)	53 (56.3)
(iii) Fireworks (*N* = 7)	0 (0.0)	0 (0.0)	7 (100.0)
*P* value	**0.2014**	**0.4484**	**0.0713**
(5) Extent of the wound			
(i) ≤10 mm (*N* = 80)	21 (26.3)	12 (15.0)	47 (58.7)
(ii) >10 mm (*N* = 26)	7 (26.9)	5 (19.2)	14 (53.9)
*P* value	**0.999**	**0.7587**	**0.8328**
(6) Zone of injury			
(i) Zone I (*N* = 69)	21 (30.4)	8 (11.6)	40 (58.0)
(ii) Zone II (*N* = 19)	3 (15.8)	6 (31.6)	10 (52.6)
(iii) Zone III (*N* = 7)	1 (14.3)	1 (14.3)	5 (71.4)
*P* value	**0.4102**	**0.1066**	**0.7764**
(7) Lens injury			
(i) Yes (*N* = 96)	23 (24.0)	21 (21.9)	52 (54.1)
(ii) No (*N* = 69)	17 (24.6)	7 (10.1)	45 (65.3)
*P* value	**0.999**	**0.0768**	**0.2069**
(8) Iris affected			
(i) Yes (*N* = 89)	18 (20.2)	17 (19.1)	54 (60.7)
(ii) No (*N* = 76)	22 (28.9)	11 (14.5)	43 (56.6)
*P* value	**0.2623**	**0.5611**	**0.7084**
(9) Hyphema			
(i) Yes (*N* = 66)	21 (31.8)	12 (18.2)	33 (50.0)
(ii) No (*N* = 99)	19 (19.2)	16 (16.2)	64 (64.6)
*P* value	**0.0952**	**0.8989**	**0.0871**
(10) Density of VH			
(i) Partial view (*N* = 39)	2 (5.1)	6 (15.4)	31 (79.5)
(ii) No view (*N* = 123)	36 (29.3)	22 (17.9)	65 (52.8)
*P* value	**0.0039** ^*^	**0.9069**	**0.0057** ^*^
(11) PPV performed			
(i) Yes (*N* = 98)	28 (28.6)	17 (17.3)	53 (54.1)
(ii) No (*N* = 67)	12 (17.9)	11 (16.4)	44 (65.7)
*P* value	**0.1663**	**0.999**	**0.1854**
(12) Lens status at last FU			
(i) Clear (*N* = 43)	8 (18.6)	4 (9.3)	31 (72.1)
(ii) Cataract (*N* = 18)	5 (27.8)	1 (5.6)	12 (66.7)
(iii) Aphakic (*N* = 66)	20 (30.3)	18 (27.3)	28 (42.4)
(iv) Pseudophakic (*N* = 31)	0 (0.0)	5 (16.1)	26 (83.9)
*P* value	**0.0012** ^*^	**0.0515**	**<0.001** ^*^

LP: light perception. HM: hand motion. CF: counting finger. *N*: number. VH: vitreous hemorrhage. PPV: pars plana vitrectomy. FU: follow-up. ^*^Statistically significant at 5% level of significance.

**Table 9:** A summary of the 18 eyes with vitreous hemorrhage caused by accidental trauma and that had retinal detachment at last follow-up.

Serial number	Eye	Age	Gender	Presentation	VA at presentation	Retina status	Management	Outcome
1	OS	11	M	Blunt trauma by door handle	NA	RD by B-scan	PPV + lensectomy	NLP TRD Pale disc
2	OS	5	F	Corneoscleral laceration Trauma by metallic hanger	CF	Flat	(1) Primary repair (2) PPV + SO + SB for RD	4/200 Inoperable TRD + PVR
3	OS	3	M	SP primary repair	NA	RD by B-scan	PPV + SB	LP Inoperable TRD
4	OD	5	F	Trauma by sharp object	NA	Flat	(1) Primary repair (2) PPV + SB	NLP Inoperable TRD + PVR
5	OD	8	M	Trauma by sharp object	LP	Flat	(1) Primary repair (2) PPV + SB	HM Inoperable TRD + PVR
6	OD	10	M	Trauma by sharp object SP primary repair	LP	RD by B-scan	PPV + SB	NLP Inoperable total RD
7	OD	7	M	SP primary repair	LP	RD by B-scan	None	NLP Macula-off TRD
8	OD	3	M	Trauma by sharp object SP primary repair	NA	RD by B-scan	PPV + SB	HM Inoperable TRD + PVR
9	OD	2	M	Trauma by sharp object SP primary repair	NA	RD by B-scan	PPV + SB + SO	NLP TRD + PVR
10	OS	4	F	Trauma by sharp object	NA	Flat	(1) Primary repair (2) PPV + SB	HM Macula-off TRD
11	OD	8	M	SP primary repair	HM	NA	PPV + SB	NLP TRD + PVR
12	OD	4	M	SP primary repair	NA	RD by B-scan	PPV + SB + SO	Poor F&F Inoperable RD
13	OD	7	M	SP primary repair	LP	RD by B-scan	PPV + SO	LP Inoperable RD
14	OS	7	M	SP primary repair	NLP	RD by B-scan	None	NLP Inoperable RD
15	OD	14	M	SP primary repair	LP	RD by B-scan	PPV + SB + SO	1/200 TRD-macular scar
16	OS	1.6	F	Trauma by sharp object	NA	Flat	(1) Primary repair (2) PPV + SB	Not F&F Macula-off TRD + PVR
17	OD	6	M	Trauma by sharp object	NA	Flat	(1) Primary repair (2) PPV + SB	CFNF TRD + PVR
18	OD	4	F	Trauma by sharp object	NA	Flat	(1) Primary repair (2) PPV + SB	Not F&F Inoperable RD

OD: right eye. OS: left eye. VA: visual acuity. F: female. M: male. SP: status post. NLP: no light perception. LP: light perception. NA: not available. RD: retinal detachment. PPV: pars plana vitrectomy. SB: scleral buckle. TRD: tractional retinal detachment. PVR: proliferative vitreoretinopathy. SO: silicon oil. CFNF: counting finger near to the face.

**Table 10:** Factors predicting retinal detachment for eyes with vitreous hemorrhage caused by accidental trauma during follow-up.

Predictor variable	Retinal detachment developed during FU	
	Yes (%)	*P* value
(1) Age at presentation		
(i) 0–3 years (*N* = 29)	6 (20.7)	**0.0209** ^*^
(ii) 4–8 years (*N* = 61)	9 (14.8)	
(iii) 9–16 years (*N* = 71)	3 (4.2)	
(2) VA at presentation		
(i) LP-HM (*N* = 66)	10 (15.2)	**0.1366**
(ii) CF (*N* = 28)	1 (3.6)	
(iii) ≥20/200 (*N* = 31)	1 (3.2)	
(3) Mechanism of trauma		
(i) Blunt (*n* = 55)	3 (5.5)	**0.1587**
(ii) Sharp/missile (*N* = 89)	13 (14.6)	
(iii) Fireworks (*N* = 7)	1 (14.3)	
(4) Wound size (open globe)		
(i) ≤10 mm (*N* = 80)	11 (13.8)	**0.999**
(ii) >10 mm (*N* = 23)	3 (13.0)	
(5) Zone of injury		
(i) Zone I (*N* = 64)	10 (15.5)	**0.7074**
(ii) Zone II (*N* = 21)	3 (14.3)	
(iii) Zone III (*N* = 8)	0 (0.0)	
(6) Lens injury		
(i) Yes (*N* = 94)	14 (14.9)	**0.1292**
(ii) No (*N* = 67)	4 (6.0)	
(7) Iris affected		
(i) Yes (*N* = 88)	12 (13.6)	**0.404**
(ii) No (*N* = 73)	6 (8.2)	
(8) Hyphema		
(i) Yes (*N* = 65)	11 (16.9)	**0.099**
(ii) No (*N* = 96)	7 (7.3)	
(9) Density of VH at presentation		
(i) Partial view (*N* = 41)	2 (4.9)	**0.1602**
(ii) No view (*N* = 117)	16 (13.7)	
(10) PPV performed		
(i) Yes (*N* = 59)	11 (18.6)	**0.043** ^*^
(ii) No (*N* = 102)	7 (6.9)	
(11) Lens at last FU		
(i) Clear (*N* = 41)	1 (2.4)	**0.0013** ^*^
(ii) Cataract (*N* = 20)	3 (15.0)	
(iii) Aphakic (*N* = 67)	14 (20.9)	
(iv) Pseudophakic (*N* = 33)	0 (0.0)	
(12) Type of trauma		
(i) Open globe (*N* = 106)	16 (15.1)	**0.0341** ^*^
(ii) Closed globe (*N* = 55)	2 (3.6)	

LP: light perception. HM: hand motion. CF: counting finger. *N*: number. VH: vitreous hemorrhage. PPV: pars plana vitrectomy. FU: follow-up. ^*^Statistically significant at 5% level of significance.

**Table 11:** A comparison between cases of vitreous hemorrhage caused by accidental trauma and nontraumatic cases.

Variable	Traumatic eyes	Nontraumatic eyes	*P* value
Total number of eyes	187	42	
Age (in years)			
(i) Mean ± SD	7.7 ± 4.0	4.9 ± 4.4	0.0004^*^
(ii) Median	7.0	3.5	
(iii) Range	0–16	0–13	
Gender (%)			
(i) M	134 (71.7)	24 (57.1)	0.0983
(ii) F	53 (28.3)	18 (42.9)	
Management (%)			
(i) PPV	104 (55.6)	12 (28.6)	0.0027^*^
(ii) Enucleation/evisceration/exenteration	3 (1.6)	5 (11.9)	0.0067^*^
RD (%)			
(i) Total number	47 (2.1)	18 (42.9)	<0.001^*^
Duration of FU in months			
(i) Mean ± SD	23.8 ± 18.9	25.8 ± 22.1	0.5490
(ii) Median	20.0	16.5	
(iii) Range	1–75	2–75	
VA at last FU	*N* = 165	*N* = 33	
(i) LP-HM (%)	40 (24.2)	11 (33.3)	0.3831
(ii) CF (%)	28 (17.0)	4 (12.1)	0.666
(iii) ≥20/200 (%)	97 (58.8)	18 (54.6)	0.7967

SD: standard deviation. PPV: pars plana vitrectomy. FU: follow-up. M: male. F: female. LP: light perception. HM: hand motion. CF: counting finger. ^*^Statistically significant at 5% level of significance.
